# Predictive value of preoperative retinal nerve fiber layer thickness for postoperative visual recovery in patients with chiasmal compression

**DOI:** 10.18632/oncotarget.19324

**Published:** 2017-07-18

**Authors:** Jing Zhang, Sunfu Zhang, Yanlin Song, Chenjing Zhu, Min He, Qingqing Ren, Baoyin Shan, Ziqiong Wang, Yunhui Zeng, Jianguo Xu

**Affiliations:** ^1^ Department of Neurosurgery, West China Hospital of Sichuan University, The First People's Hospital of Yibin, Sichuan, PR China; ^2^ West China School of Medicine, West China Hospital of Sichuan University, Sichuan, PR China

**Keywords:** retinal nerve fiber layer, optical coherence tomography, visual recovery, chiasmal compression, surgery

## Abstract

The aim of this study was to evaluate the predictive role of preoperative retinal nerve fiber layer (RNFL) thickness for postoperative visual recovery in patients with chiasmal compression through performing a meta-analysis. PubMed, EMBASE, Cochrane Library and China National Knowledge Infrastructure were searched for relevant studies. The study and patient characteristics were extracted. Pooled odds ratio (OR) with 95% confidence interval (CI) was calculated to estimate the predictive value of RNFL thickness. Subgroup analyses were also performed. Four studies with 202 patients and 395 eyes were included. The pooled results showed that patients with normal RNFL thickness could achieve better visual recovery compared with those with thin RNFL with the OR of 15.61 (95% CI, 4.09-59.61). Significant heterogeneity was observed (I^2^ = 54.5%, P=0.086). Publication bias was not present. Normal preoperative RNFL thickness could predict better postoperative visual recovery than thin RNFL in patients with chiasmal compression.

## INTRODUCTION

Sellar and parasellar region lesions, such as pituitary adenoma, cystic lesions, craniopharyngioma and meningioma, always lead to the compression of optic chiasm [1–3]. Among them, pituitary adenoma, which accounts for 12–15% of all intracranial lesions, is the most common [4]. As a consequence, visual impairment, including visual field (VF) defects and loss of visual acuity (VA), is a common complaint in these patients [3, 5]. Surgical removal of the lesions is an important aspect of clinical management. Although some patients could gain an appreciable visual recovery after surgical decompression, a great number of them could not [6]. Many researchers have investigated potential predictive factors, and variables such as age, size of tumor, preoperative visual impairment, preoperative duration of symptoms and optic atrophy were suggested [7–9]. However, conflicting results still remain, and none of them were regarded as reliable predictors for visual recovery [10, 11].

Recently, retinal nerve fiber layer (RNFL) thickness was proposed to predict postoperative visual recovery in patients with chiasmal compression [3, 12]. The thinning of RNFL, which is composed of retinal ganglion cell (RGC) axons, could reflect axonal degeneration of RGC [11, 13]. Irreversible damage imposed to RGC axons by longstanding compression before surgery is thought to result in poor visual outcome after surgery [10, 11]. Optical coherence tomography (OCT) is a rapid, objective, quantitative, non-invasive method to measure the RNFL thickness [11, 14]. Some researchers investigated the prognostic role of RNFL thickness for visual recovery in patients with pituitary adenomas [14, 15], while others focused on parachiasmal meningioma and other tumors [3, 11]. The aim of this study was to systematically evaluate the predictive value of RNFL thickness for visual recovery in patients with chiasmal compression through performing a meta-analysis.

## RESULTS

### Literature research

**Figure 1:**
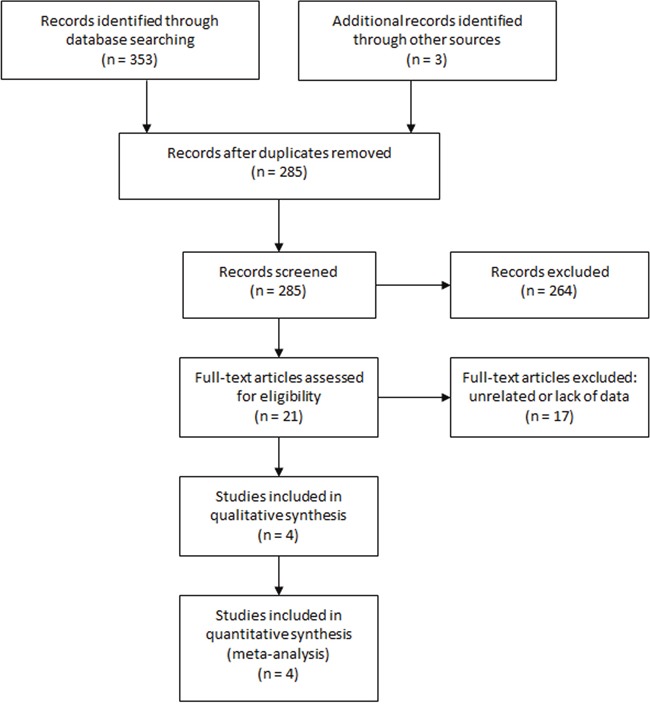
Selection process of studies

The initial literature search identified 356 studies. After removing duplicates, 285 studies were reviewed for titles and abstracts. Six reviews, 13 case reports and 245 unrelated articles were excluded. The rest 21 studies were assessed in full text and 17 were excluded due to unrelated or lack of enough data. Finally, 4 studies [3, 11, 14, 15] were included in this meta-analysis. The study selection process was shown in Figure 1.

### Study characteristics

The main characteristics of the 4 included studies were shown in Table 1. The patients in two of the four studies were from Asia. Three studies were prospective and one was retrospective. The total number of patients was 202 (range from 25 to 107) with 395 eyes (range from 49 to 213) investigated. All the patients were diagnosed with chiasmal compression and were treated with surgery. Three studies used visual field examination as visual function evaluating tool while one study used visual acuity examination. Three studies had a follow-up time of more than 3 months and one study assessed postoperative visual function within 6 weeks after surgery. All the studies concluded that normal RNFL thickness predicted better visual recovery.

**Table 1:** Characteristics of the included studies

First author	Year	Study design	Patient source	N (F/M)	Mean age	No. of eyes	Normal RNFL	Thin RNFL	Tumor type	Surgical approach	RNFL cut-off^#^	Vision test	Follow- up time	OR adjusted	Attitude
Danesh-Meyer	2008	prospective	New Zealand/USA	35 (18/17)	45	63	43	20	mixed*	not reported	97.5%	VF	≤6W	no	positive^&^
Danesh-Meyer	2015	prospective	Australia	107 (49/58)	53.67	213	178	35	PA	both	95%	VA	9–15M	no	positive
Park	2015	retrospective	Korea	25 (23/2)	51.5	49	28	21	meningioma	transcranial	95%	VF	3.1–31.7M	no	positive
Yoneoka	2015	prospective	Japan	35 (13/22)	57.6	70	45	25	PA	transsphenoidal	99%	VF	≥3M	yes	positive

N (F/M) number of patients (Female/Male), RNFL retinal nerve fiber layer, PA pituitary adenoma, VF visual field, VA visual acuity, W weeks, M months, OR odds ratio.

* mixed: 27 pituitary adenoma, 3 cystic lesions, 2 meningioma, 1 craniopharyngioma, 1 neurosarcoidosis, 1 paraclinoid aneurysm;

^#^ RNFL cut-off: thinner than 99%/97.5%/95% of normative values obtained from the manufacturer's database;

^&^ positive: normal RNFL thickness predicted better visual recovery.

### Overall analysis

**Figure 2:**
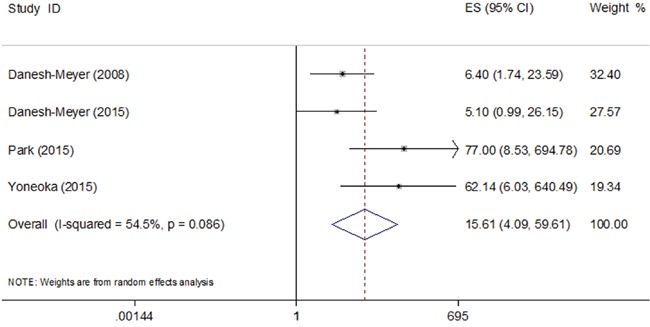
Pooled odds ratio (OR) of visual recovery in patients with normal RNFL thickness compared with those with thin RNFL

The pooled results of the four studies showed that eyes with normal RNFL thickness could achieve better visual recovery compared with those with thin RNFL with the OR of 15.61 (95% CI, 4.09-59.61) (Figure 2). Significant heterogeneity was observed (I^2^ = 54.5%, P=0.086). Sensitivity analysis identified the study by Park et al. [11] to be a significant contributor to the heterogeneity. After excluding this study, the heterogeneity shrinked to 40.3% (P=0.187) and the pooled OR remained statistically significant (OR 8.56; 95% CI, 3.36-21.78).

### Subgroup analysis

The pooled ORs in the Asian and non-Asian group were 69.61 (95% CI, 14.05-344.93) and 5.86 (95% CI, 2.11-16.24), respectively. The pooled OR of the three prospective studies was 8.56 (95% CI, 3.36-21.78) compared to the OR of 77.00 (95% CI, 8.53-694.81) in the retrospective study. The pooled OR of the three studies that used visual field examination as visual function evaluating tool was 25.38 (95% CI, 4.31-149.65) compared to the OR of 5.10 (95% CI, 1.00-26.30) in the study that used visual acuity examination. And the pooled OR of the three studies that had a follow-up time of more than 3 months was 25.12 (95% CI, 3.88-162.67) compared to the OR of 6.40 (95% CI, 1.70-23.10) in the study with the follow-up time within 6 weeks after surgery.

The patients in the two studies by Danesh-Meyer et al. [14] and Yoneoka et al. [15] were all with pituitary adenomas and were treated with transsphenoidal surgery except one with transcranial surgery. We grouped the two studies into pituitary adenomas group/transsphenoidal group, and the rest two into mixed tumors group/non-transsphenoidal group. The pooled OR in the pituitary adenomas group/transsphenoidal group was 15.41 (95% CI, 1.35-175.70) and the pooled OR in the mixed tumors group/non-transsphenoidal group was 18.84 (95% CI, 1.68-211.09).

All the pooled results were shown in Table 2.

**Table 2:** Summary of meta-analysis results

	N	Model	Pooled OR (95% CI)	P value	Heterogeneity(P, I^2^)	Publication bias	Conclusion
Total	4	Random	15.61 (4.09-59.61)	<0.001	0.086, 54.5%	0.308	Positive
Asian	2	Fixed	69.61 (14.05-344.93)	<0.001	0.896, 0.0%	1.000	Positive
Non-Asian	2	Fixed	5.86 (2.11-16.24)	0.001	0.831, 0.0%	1.000	Positive
Prospective	3	Fixed	8.56 (3.36-21.78)	<0.001	0.187, 40.3%	1.000	Positive
Retrospective	1	—	77.00 (8.53-694.81)	<0.05	—	—	Positive
VF	3	Random	25.38 (4.31-149.65)	<0.001	0.076, 61.2%	1.000	Positive
VA	1	—	5.10 (1.00-26.30)	0.034	—	—	Positive
Follow-up ≥ 3 months	3	Random	25.12 (3.88-162.67)	0.001	0.081, 60.2%	1.000	Positive
Follow-up < 3 months	1	—	6.40 (1.70-23.10)	0.007	—	—	Positive
Pituitary adenoma	2	Random	15.41 (1.35-175.70)	0.028	0.085, 66.2%	1.000	Positive
Other tumor types	2	Random	18.84 (1.68-211.09)	0.017	0.057, 72.5%	1.000	Positive
Transsphenoidal	2	Random	15.41 (1.35-175.70)	0.028	0.085, 66.2%	1.000	Positive
Transcranial/unknown	2	Random	18.84 (1.68-211.09)	0.017	0.057, 72.5%	1.000	Positive

VF visual field, VA visual acuity, N number of included studies, OR odds ratio, CI, confidence interval.

### Publication bias

**Figure 3:**
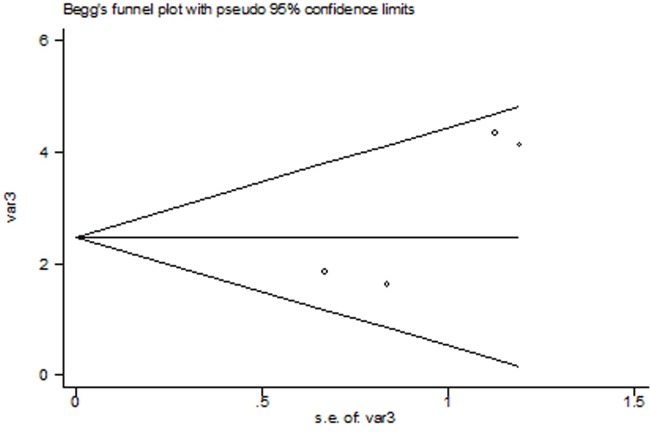
The Begg's publication bias plot of the 4 included studies

The Begg's publication bias plot of the 4 included studies was shown in Figure 3, and publication bias was not found (P= 0.308).

## DISCUSSION

### Implications

This study aimed to investigate the predictive value of RNFL thickness for visual recovery in patients with chiasmal compression. A meta-analysis was performed to pool the existing evidence and four studies were included. This meta-analysis showed that patients with normal preoperative RNFL thickness could achieve better visual recovery than patients with thin RNFL.

Subgroup analyses were also performed to examine the role of RNFL thickness. The pooled OR in the Asian group was much higher than that in the non-Asian group, demonstrating that the predictive value of RNFL thickness for visual recovery might be stronger in Asian population than non-Asian population. The OR in the only one retrospective study was much higher than the pooled OR of the three prospective studies, and that could be explained that the retrospective study might have potential selection bias. The pooled OR of the three studies that used visual field examination as visual function evaluating tool was higher than the OR of the study that used visual acuity examination, showing that RNFL thickness might better predict visual field recovery than visual acuity recovery. The pooled OR of the three studies that had a follow-up time of more than 3 months was higher than the study with the follow-up time within 6 weeks, suggesting that RNFL thickness might have a stronger predictive value for long term visual recovery. Some researchers found that most visual recovery occurs within 3 months [14], even within 2 weeks [15]. Lee et al. [10] found that the visual field did not recovery significantly between 3 and 6 months after surgery. So our results revealed that normal RNFL thickness might lead to better long term visual recovery. We also found that the pooled OR in the pituitary adenomas group/transsphenoidal group was almost the same as that in the mixed tumors group/non-transsphenoidal group. As we know, there is little surgical trauma via transsphenoidal approach [11]. Our results showed that RNFL thickness could well predict visual recovery no matter whether there was much surgical trauma to the visual pathway or not. However, one thing that should be paid attention to is that our study was based on only four studies, so our conclusions from subgroup analyses are still open to discuss.

Despite the included studies, Jacob et al. [18] found that, for each increase by 1μm of mean RNFL, the odds of complete recovery was multiplied by 1.29 (P=0.037) three months after the initial VF defect. Also, Garcia et al. [19] found that nasal RNFL thickness was a good prognostic factor for VF recovery with the OR of 1.56 per 15 μm (P=0.041). Furthermore, Avery et al. [20] suggested the use of RNFL thickness measurement as a biomarker of vision in children who could not cooperate for VA/VF testing. Since the thinning of RNFL reflects axonal degeneration of RGC, undelayed surgical decompression before axonal loss is desired. Maybe in the future, with the evidence accumulates, the time for surgical treatment rely on the preoperative RNFL thickness even in asymptomatic patients with chiasmal compression.

Average RNFL thickness of the superior, inferior, temporal and nasal quadrants RNFL thicknesses was widely used to predict visual recovery. Some researchers also explored the predictive value of RNFL thicknesses in different quadrants. Jacob et al. [18] found that inferior RNFL thicknesses was a strong prognostic factor. Garcia et al. [19] favored nasal RNFL thicknesses since nasal axons decussate at the optic chiasm. More studies are needed to find out which quadrant of RNFL could better predict visual recovery.

Nevertheless, a thin RNFL could not preclude visual recovery and RNFL thickness is not the only marker for visual recovery. Other factors, such as age, preoperative duration of symptoms, optic atrophy, size of tumor, adjacent tissue adherence of tumor [21], and optic canal invasion [11] are also important in visual recovery. Considering that, Lee et al. [10] constructed a nomogram to predict restoration of the visual field in patients with pituitary adenomas using MRI compression grade, preoperative VF, and preoperative inferior RNFL thickness. The area under the curve (AUC) of their nomogram was 0.84. More well-designed studies are warranted to establish a good predictive model for visual recovery after decompressive surgery to allow a tailored counseling of individual patients.

Significant heterogeneity was present in this meta-analysis and sensitivity analysis identified the study by Park et al. [11] to be the major contributor to the heterogeneity. The heterogeneity reduced to 40.3% after the exclusion of this study (P=0.187) and the pooled OR remained statistically significant. The reason why this study contributed to heterogeneity might be that it was the only retrospective study and its result was affected by some potential bias. Other sources of heterogeneity might be from different patient sources, tumor types, surgical approaches, and visual function evaluating tools.

### Limitations

There are some limitations in this study. Firstly, our meta-analysis was based on a limited number of studies. And the results in the subgroup analyses were not definitely accurate. Secondly, the cut-off values of RNFL thickness in the included studies varied. More studies are needed to address the best cut-off value to predict visual recovery. Furthermore, significant heterogeneity was present and random effects model was used. Besides, publication bias was a major concern for all meta-analyses. No publication bias was found in our study, but it should not be completely excluded.

## MATERIALS AND METHODS

### Search strategy

We followed the developed guidelines for systematic reviews and meta-analyses in performing our study [16]. PubMed, EMBASE, Cochrane Library and China National Knowledge Infrastructure (CNKI) were searched for potentially relevant literature (last search ran on Nov 16th, 2016). The following search terms were used: (chiasmal compression OR optic chiasm compression OR parachiasmal OR optic chiasm OR sellar OR parasellar OR pituitary neoplasms OR craniopharyngioma OR parachiasmal meningioma) AND (retinal nerve fiber layer OR optical coherence tomography) AND (vision OR visual). Reference lists of selected articles were also screened for any additional studies. No language restrictions were used.

### Study selection

The study selection process was performed by two reviewers (J.Z. and Z.W.) independently with any discrepancies being discussed. Studies were included according to the following inclusion criteria: (1) the patients were diagnosed with chiasmal compression and surgery was indicated; (2) preoperative RNFL thickness and visual function were assessed in patients; (3) postoperative visual function was also assessed; (4) enough data were reported to estimate the prognostic role of preoperative RNFL thickness in postoperative visual recovery. Visual recovery was defined as the improvement of visual acuity or visual function. Reviews, letters, meeting abstracts, case reports, unrelated articles, and studies without enough data were excluded.

### Data extraction

Two independent researchers (J.Z. and Y.S.) extracted the data from the included studies and disagreements were resolved by consensus. Odds ratio (OR) with 95% confidence interval (CI), or the data that could be used to calculate the OR with 95% CI was extracted as primary data. If adjusted OR and unadjusted OR both existed, adjusted OR was used. The study and patients characteristics included first author, publication year, patient source, study design, number of samples, patients’ mean age and sex, tumor type, surgical details, the machine type used to measure RNFL thickness, the cut-off value of RNFL thickness, visual function evaluating tools, follow up time and study attitude.

### Statistical analysis

As to the studies without OR or 95% CI, OR and 95% CI were calculated using the patients data by RevMan 5.1 (Cochrane collaboration, Oxford, UK). The logOR and variance were then calculated and used for aggregation of the prognostic role of RNFL thickness. The data was pooled together by STATA 11.0 (STATA Corporation, College Station, TX). Forest plots were used to estimate the pooled OR. The pooled OR was considered significant if the 95% CI did not overlap 1 and the P value was less than 0.05. Subgroup analyses based on patient source, study design, tumor type, surgical details, visual function evaluating tools and follow up time were performed. The between-study heterogeneity was assessed and significant heterogeneity was considered present with P value<0.10 or I^2^>50% [17]. A random effect model was used if heterogeneity existed. And sensitivity analysis was performed to examine the contribution of each study to heterogeneity by excluding individual studies one at a time when heterogeneity was present. Publication bias was assessed by Begg's test with P<0.05 indicating potential publication bias. Heterogeneity assessment and publication bias assessment were performed by STATA 11.0.

## CONCLUSION

In conclusion, our study suggested that normal preoperative RNFL thickness could predict better postoperative visual recovery than thin RNFL in patients with chiasmal compression. These findings could help the management of the timing of surgery, counseling patients on prognosis, and even monitoring disease progression. More studies are needed to corroborate these findings and address the predictive value of each quadrant of RNFL. Moreover, studies are warranted to establish a model that takes account various factors to predictive visual recovery.

## References

[1] Tagoe NN, Essuman VA, Fordjuor G, Akpalu J, Bankah P, Ndanu T (2015). Neuro-Ophthalmic and Clinical Characteristics of Brain Tumours in a Tertiary Hospital in Ghana. Ghana Med J.

[2] Seeburg DP, Dremmen MH, Huisman TA (2017). Imaging of the Sella and Parasellar Region in the Pediatric Population. Neuroimaging Clin N Am.

[3] Danesh-Meyer HV, Papchenko T, Savino PJ, Law A, Evans J, Gamble GD (2008). *In vivo* retinal nerve fiber layer thickness measured by optical coherence tomography predicts visual recovery after surgery for parachiasmal tumors. Invest Ophthalmol Vis Sci.

[4] Anderson D, Faber P, Marcovitz S, Hardy J, Lorenzetti D (1983). Pituitary tumors and the ophthalmologist. Ophthalmology.

[5] Phal PM, Steward C, Nichols AD, Kokkinos C, Desmond PM, Danesh-Meyer H, Sufaro YZ, Kaye AH, Moffat BA (2016). Assessment of Optic Pathway Structure and Function in Patients With Compression of the Optic Chiasm: A Correlation With Optical Coherence Tomography. Invest Ophthalmol Vis Sci.

[6] Laws ER, Trautmann JC, Hollenhorst RW (1977). Transsphenoidal decompression of the optic nerve and chiasm Visual results in 62 patients. J Neurosurg.

[7] Yu FF, Chen LL, Su YH, Huo LH, Lin XX, Liao RD (2015). Factors influencing improvement of visual field after trans-sphenoidal resection of pituitary macroadenomas: a retrospective cohort study. Int J Ophthalmol.

[8] Ho RW, Huang HM, Ho JT (2015). The influence of pituitary adenoma size on vision and visual outcomes after trans-sphenoidal adenectomy: a report of 78 cases. J Korean Neurosurg Soc.

[9] Gnanalingham KK, Bhattacharjee S, Pennington R, Ng J, Mendoza N (2005). The time course of visual field recovery following transphenoidal surgery for pituitary adenomas: predictive factors for a good outcome. J Neurol Neurosurg Psychiatry.

[10] Lee J, Kim SW, Kim DW, Shin JY, Choi M, Oh MC, Kim SM, Kim EH, Kim SH, Byeon SH (2016). Predictive model for recovery of visual field after surgery of pituitary adenoma. J Neurooncol.

[11] Park HH, Oh MC, Kim EH, Kim CY, Kim SH, Lee KS, Chang JH (2015). Use of optical coherence tomography to predict visual outcome in parachiasmal meningioma. J Neurosurg.

[12] Fraser CL, Biousse V, Newman NJ (2012). Visual outcomes after treatment of pituitary adenomas. Neurosurg Clin N Am.

[13] Kanamori A, Catrinescu MM, Belisle JM, Costantino S, Levin LA (2012). Retrograde and Wallerian axonal degeneration occur synchronously after retinal ganglion cell axotomy. Am J Pathol.

[14] Danesh-Meyer HV, Wong A, Papchenko T, Matheos K, Stylli S, Nichols A, Frampton C, Daniell M, Savino PJ, Kaye AH (2015). Optical coherence tomography predicts visual outcome for pituitary tumors. J Clin Neurosci.

[15] Yoneoka Y, Hatase T, Watanabe N, Jinguji S, Okada M, Takagi M, Fujii Y (2015). Early morphological recovery of the optic chiasm is associated with excellent visual outcome in patients with compressive chiasmal syndrome caused by pituitary tumors. Neurol Res.

[16] Moher D, Liberati A, Tetzlaff J, Altman DG (2009). Preferred reporting items for systematic reviews and meta-analyses: the PRISMA statement. J Clin Epidemiol.

[17] Higgins JP, Thompson SG, Deeks JJ, Altman DG (2003). Measuring inconsistency in meta-analyses. BMJ.

[18] Jacob M, Raverot G, Jouanneau E, Borson-Chazot F, Perrin G, Rabilloud M, Tilikete C, Bernard M, Vighetto A (2009). Predicting visual outcome after treatment of pituitary adenomas with optical coherence tomography. Am J Ophthalmol.

[19] Garcia T, Sanchez S, Litre CF, Radoi C, Delemer B, Rousseaux P, Ducasse A, Arndt C (2014). Prognostic value of retinal nerve fiber layer thickness for postoperative peripheral visual field recovery in optic chiasm compression. J Neurosurg.

[20] Avery R, Hwang E, Acosta M, Hutcheson K, Santos D, Zand D, Kilburn L, Rosenbaum K, Rood B, Packer R (2012). Hand-held optical coherence tomography during sedation detects visual acuity and visual field loss in young children with optic pathway gliomas. Neuro-Oncology.

[21] Rosenberg LF, Miller NR (1984). Visual results after microsurgical removal of meningiomas involving the anterior visual system. Arch Ophthalmol.

